# New Insights into Foodborne Bacteria–Host Interactions: Evolving Research and Discoveries

**DOI:** 10.3390/microorganisms12010078

**Published:** 2023-12-30

**Authors:** Anja Klančnik, Maja Abram

**Affiliations:** 1 Department of Food Science and Technology, Biotechnical Faculty, University of Ljubljana, 1000 Ljubljana, Slovenia; 2 Department of Microbiology and Parasitology, Faculty of Medicine, University of Rijeka, 51000 Rijeka, Croatia; maja.abram@medri.uniri.hr; 3 Department of Clinical Microbiology, Clinical Hospital Centre Rijeka, 51000 Rijeka, Croatia

Given the growing concern about foodborne diseases, intensive research and the development of new approaches are crucial. As shown in Table 1, recent scientific advances in the fight against foodborne pathogens and intestinal diseases have led to various innovative approaches. In the area of poultry health, groundbreaking vaccine research against *Clostridium perfringens* marks a significant shift from relying on antibiotics to more sustainable practices. In the area of food safety, a study on the role of raw sheep’s milk in salmonella outbreaks highlights the critical link between milk processing and public health and highlights the need for stringent safety standards in the dairy industry. In the field of probiotics, the particular focus on *Bifidobacterium longum* helps to improve our understanding of the gut microbiome and develop more effective probiotic therapies. At the same time, alternative treatments for *Campylobacter jejuni* infections are being explored, including clove essential oil, which has been shown to reduce bacterial load and inflammation without antibiotics. This is part of a broader strategy that combines natural remedies with probiotic findings for a comprehensive treatment of intestinal infections. In addition, reviews on microbial biofilm formation and antibiotic resistance emphasize the urgent need for integrated strategies to improve food safety. These studies demonstrate the interdisciplinary nature of current research and emphasize the importance of combining different scientific approaches to effectively combat foodborne pathogens. The study by Fu et al. (Table 1) addresses a critical problem in poultry health: necrotic enteritis caused by *Clostridium perfringens*. The groundbreaking contribution of this research lies in the development of vaccines using the bacterium’s sporulation proteins. Until now, antibiotics have been used to combat this disease, but their overuse has led to an alarming increase in antibiotic resistance, which poses a significant global health risk. This innovative vaccine strategy could revolutionize poultry health management and promise to improve poultry welfare and align with global efforts to minimize antibiotic use in animal husbandry, leading to healthier poultry production and safer products for human consumption.

The study by Napoleoni et al. (Table 1), looking at the dairy industry, examines the public health risks associated with consuming raw sheep’s milk cheese. By meticulously tracing an outbreak of *Salmonella enteritidis* in central Italy to unpasteurized dairy products, this study highlights the urgent need for strict food safety and hygiene standards in dairy production. It draws attention to the vulnerability of dairy supply chains to bacterial contamination and the resulting public health consequences and emphasizes the importance of comprehensive safety protocols and consumer education about the risks of raw milk products. This study is an important link between agricultural practices and public health and highlights the importance of food safety in the dairy sector.

In the field of gut health, a notable step forward has been made through a study by Xiao et al. (Table 1) using bioinformatics to identify specific strains of *Bifidobacterium longum* in stool samples. This research goes beyond the usual species-level analysis of probiotics and explores the complex strain-specific interactions within the gut microbiome. By distinguishing the unique roles of different strains, this approach deepens our understanding of gut colonization and the efficacy of probiotics, ushering in a new era of personalized nutrition and gut health management that has the potential for tailored probiotic therapies.

In the study by Weschka et al. (Table 1) Aviguard, a probiotic formulation, was tested for its efficacy against *C. coli* infections in mice. While the formulation had limited success in reducing the pathogen load in the distal gut, it showed considerable potential in modulating the immune response and reducing inflammation. This discovery opens up new possibilities for using probiotics in treating intestinal infections as potential therapeutics that could complement or serve as an alternative to antibiotics, which is particularly important in the face of increasing antibiotic resistance. In addition to these results, the study by Šimunović et al. [1] underlines the effectiveness of probiotics for poultry health. These results underscore the potential of *B. subtilis* PS-216 as an effective probiotic intervention in poultry production, contributing to safer poultry meat and improved animal health.

Bereswill et al. (Table 1) present clove essential oil as a promising natural remedy for treating *C. jejuni* infections and show that it can reduce bacterial load and inflammation in a mouse model, offering an innovative, antibiotic-free approach to campylobacteriosis treatment. This research is in line with previous studies, such as that of Klančnik et al. [2], which highlighted the anti-adhesion effect of phytochemicals against *C. jejuni*. In an era of increasing antibiotic resistance, research into natural substances such as clove essential oil is crucial for developing effective, sustainable, and accessible alternatives to conventional antibiotics to control foodborne diseases.

The review by Oktariani et al. (Table 1) deals with histamine production in seafood, which is mainly caused by histamine-producing bacteria (HPB). It highlights the risks associated with histamine ingestion, such as food poisoning and allergenic reactions. The study highlights the methods for detecting HPB and controlling histamine levels in seafood and recommends temperature control and freezing as effective strategies. This emphasizes the need for strict safety measures when handling and processing seafood.

The focus in the review by Nadar et al. (Table 1) then shifts to biofilms, complex microbial communities that contribute significantly to antibiotic resistance. Innovative biofilm agents and strategies will be explored, emphasizing the importance of targeting biofilm formation to effectively combat resistant microbial strains. Various biofilm agents, including small molecules, antimicrobial peptides, and natural products, will be investigated for their potential to disrupt biofilms and improve the efficacy of existing antibiotics.

By synthesizing the findings from all studies (Table 1), the summary presents a multi-faceted approach to understanding and managing microbial threats. It improves our understanding of food safety, particularly in seafood and poultry, and addresses the public health implications of microbial contamination. We also provide an overview of research published in various MDPI journals and in the journal *Microorganisms* that focuses on the critical area of foodborne pathogens. Key topics (i.e., foodborne) include antimicrobial resistance, the development of biosensors, epidemiology, and advanced detection methods, with a particular focus on the growing concern over antibiotic resistance. These topics, covered in over 500 articles, reveal a dynamic, evolving field and scientific community grappling with the complexities of foodborne pathogens. The shift towards technological innovation in pathogen detection reflects a proactive approach to food safety research [3].

Of particular note is this Special Issue of *Microorganisms* [4], which deals with *Campylobacter*, an important human pathogen, and uses the latest research findings to show significant progress in understanding and combating this pathogen. This Special Issue focuses on *Campylobacter jejuni*, a major cause of bacterial gastroenteritis worldwide, with an emphasis on antibiotic resistance. The transcriptomic analysis by Kovács et al. [5] focuses on the virulence characteristics of *C. jejuni*, while Talukdar et al. [6] and Guirado et al. [7] examine the interactions of virulence proteins with host cells and gene variations associated with Guillain–Barré syndrome, respectively. Other research, such as [8] and [9,10], investigates new infection models and potential treatment options for *C. jejuni*-induced enterocolitis. Rapp et al. [11] investigate the environmental and wildlife factors in *Campylobacter* transmission. Research on *C. coli* in pigs focuses on prevalence and resistance as well as on the development of storage and transportation methods for bacteriophages. In addition, Šimunović et al. [12] compare *C. jejuni* from abattoirs and surface water and emphasize its better adaptation to the chicken host environment. This research expands our understanding of pathogen behavior and provides information for strategies to manage foodborne pathogens. Incorporating in silico bioinformatics, Darwin et al. [13] use machine learning and population genomics to improve our understanding of *Campylobacter* pathogenicity. The papers in this Special Issue, including a systematic analysis of vaccine candidates against *C. jejuni* for chickens and studies on biofilm formation, contribute to a comprehensive understanding of the role of *Campylobacter* in public health and food safety.

Based on these findings, the importance of improved safety protocols in food handling and processing is further emphasized. Advances in biofilm research, including studies on the surfactome by Janež et al. [14] on microbial interactions by Klančnik et al. [9], and on new functions of signaling molecules by Ramić et al. [15] have highlighted new directions in this field. These developments are crucial for developing targeted therapeutic strategies against resistant strains and thus for improving food safety standards. The studies in this Special Issue focus on foodborne pathogens and enteric diseases using various strategies, including developing vaccines targeting bacterial proteins, advanced bioinformatics in probiotics, and stringent food safety measures. A major focus is on emerging pathogens such as *Campylobacter*, a major cause of campylobacteriosis [16], as well as the study of protein-glycan interactions and biofilm structures [17]. This research paves the way for stricter standards in food production and innovative solutions to microbial infections and emphasizes the interdisciplinary nature of tackling food safety and public health challenges.

Overall, these studies make important contributions to the development of public health strategies to prevent foodborne diseases and are crucial for designing food safety policies and regulations, for new treatment methods, and for raising awareness of foodborne pathogens.

## Figures and Tables

**Table 1:** Analysis of the published contributions in this Special Issue.

N#	*Type* Title	Authors	Research Area	Focus	Conclusions
1	*Research:* Vaccines Using *Clostridium perfringens* Sporulation Proteins Reduce Necrotic Enteritis in Chickens	Fu et al.	Veterinary Medicine, Microbiology	Vaccination against *Clostridium perfringens* in chickens to reduce necrotic enteritis	Vaccines using sporulation proteins of *C. perfringens* are effective in reducing necrotic enteritis in chickens and emphasize the importance of sporulation in the pathogenesis
2	*Research:* A Strong Evidence Outbreak of *Salmonella enteritidis* in Central Italy Linked to the Consumption of Contaminated Raw Sheep Milk Cheese	Napoleoni et al.	Public Health, Epidemiology	Outbreak of *Salmonella Enteritidis* linked to raw sheep milk cheese	*S. Enteritidis* in sheep’s milk poses a significant risk to public health when used to produce raw milk cheese, as the outbreak in central Italy has shown.
3	*Research:* Quantitative Detection of *Bifidobacterium longum* Strains in Feces Using Strain-Specific Primers	Xiao et al.	Microbiology, Biotechnology	Development of strain-specific primers for quantitative detection of *B. longum* in fecal samples	Strain-specific primers are effective for the quantitative detection of *B. longum* strains in feces and provide insights into the dynamics of intestinal colonization.
4	*Research:* Survey of Pathogen-Lowering and Immuno-Modulatory Effects Upon Treatment of *Campylobacter coli*-Infected Secondary Abiotic IL-10−/− Mice with the Probiotic Formulation Aviguard^®^	Weschka et al.	Immunology, Veterinary Medicine	The effects of Aviguard^®^ on *Campylobacter coli* infection in mice	Aviguard^®^ reduces the pathogen load in the proximal intestinal tract and modulates the immune response during *C. coli* infection in mice.
5	*Research:* Peroral Clove Essential Oil Treatment Ameliorates Acute Campylobacteriosis—Results from a Preclinical Murine Intervention Study	Bereswill et al.	Pharmacology, Microbiology	The effect of clove essential oil on acute campylobacteriosis in mice	Clove essential oil shows potential as a natural, antibiotic-independent treatment for acute campylobacteriosis with antimicrobial and immunomodulatory effects.
6	*Review*: Role of Marine Bacterial Contaminants in Histamine Formation in Seafood Products	Oktariani et al.	Food Safety, Marine Biology	Histamine production in seafood by marine bacteria, food safety, methods to control histamine formation	Marine bacteria play a key role in histamine production in seafood, which can lead to food poisoning. Control methods include temperature control and freezing.
7	*Review:* Development of Antibiofilm Therapeutics Strategies to Overcome Antimicrobial Drug Resistance	Nadar et al.	Microbiology, Pharmacology	Biofilm formation in pathogenic bacteria, strategies to control biofilm formation, antibiofilm agents	New strategies and agents that target biofilms in pathogenic bacteria are essential for overcoming antibiotic resistance. These include photodynamic therapy, antibodies, macrophages, and nanoparticle systems.
